# The mitochondrial genome of the 'twisted-wing parasite' *Mengenilla australiensis *(Insecta, Strepsiptera): a comparative study

**DOI:** 10.1186/1471-2164-10-603

**Published:** 2009-12-14

**Authors:** Dino P McMahon, Alexander Hayward, Jeyaraney Kathirithamby

**Affiliations:** 1 Department of Zoology, University of Oxford, The Tinbergen Building, South Parks Road, Oxford, OX1 3PS, UK

## Abstract

**Background:**

Strepsiptera are an unusual group of sexually dimorphic, entomophagous parasitoids whose evolutionary origins remain elusive. The lineage leading to *Mengenilla australiensis *(Family Mengenillidae) is the sister group to all remaining extant strepsipterans. It is unique in that members of this family have retained a less derived condition, where females are free-living from pupation onwards, and are structurally much less simplified. We sequenced almost the entire mitochondrial genome of *M. australiensis *as an important comparative data point to the already available genome of its distant relative *Xenos vesparum *(Family Xenidae). This study represents the first in-depth comparative mitochondrial genomic analysis of Strepsiptera.

**Results:**

The partial genome of *M. australiensis *is presented as a 13421 bp fragment, across which all 13 protein-coding genes (PCGs), 2 ribosomal RNA (rRNA) genes and 18 transfer RNA (tRNA) sequences are identified. Two tRNA translocations disrupt an otherwise ancestral insect mitochondrial genome order. A+T content is measured at 84.3%, C-content is also very skewed. Compared with *M. australiensis*, codon bias in *X. vesparum *is more balanced. Interestingly, the size of the protein coding genome is truncated in both strepsipterans, especially in *X. vesparum *which, uniquely, has 4.3% fewer amino acids than the average holometabolan complement. A revised assessment of mitochondrial rRNA secondary structure based on comparative structural considerations is presented for *M. australiensis *and *X. vesparum*.

**Conclusions:**

The mitochondrial genome of *X. vesparum *has undergone a series of alterations which are probably related to an extremely derived lifestyle. Although *M. australiensis *shares some of these attributes; it has retained greater signal from the hypothetical most recent common ancestor (MRCA) of Strepsiptera, inviting the possibility that a shift in the mitochondrial selective environment might be related to the specialization accompanying the evolution of a small, morphologically simplified completely host-dependent lifestyle. These results provide useful insights into the nature of the evolutionary transitions that accompanied the emergence of Strepsiptera, but we emphasize the need for adequate sampling across the order in future investigations concerning the extraordinary developmental and evolutionary origins of this group.

## Background

Strepsiptera are an unusual group of obligate endoparasitoid insects [1]. They occur as a small (approx. 600 spp.) monophyletic insect order with uncertain evolutionary origins, inclusive of any clear understanding over the group's nearest extant relative. Strepsiptera parasitize 7 orders of insects, including silverfish (Thysanura); cockroaches (Blattaria); mantids (Mantodea); crickets and grasshoppers (Orthoptera); bugs (Hemiptera); wasps, ants and bees (Hymenoptera) and flies (Diptera). Understandably, the very uniqueness of Strepsiptera makes their placement within insects using morphological taxonomic methods a contentious task. Strepsiptera have been placed as the sister-group to myriad different insect groups, from beetles to true flies, even being placed outside of Holometabola: each hypothesis being founded on one or two 'key' characteristics that at one time or another have come under question [2-15]. Confident assertions of classification have especially been restricted because intermediate forms have largely gone extinct and are unrecorded in the fossil record (see [16] for a notable exception). Simplification of gross morphology during strepsipteran evolutionary specialization can also be viewed as a significant component of this problem: observable morphological variation is low and unevenly distributed between extremely dimorphic sexes.

Females are especially strongly simplified (lacking in most typical adult characters such as wings, legs or mouthparts), and in most strepsipteran species, they remain in the living host until the end of the reproductive cycle. Conversely, males metamorphose in a typical holometabolan fashion; developing wings and a usual suite of adult insect characters (such as antennae, mouthparts and compound eyes) only to leave the host immediately in search of a mate; usually in the form of a female-containing host [17-19]. Males deliver sperm through a brood canal opening in the cephalothorax (the modified "head") of the female, who as a reproductive adult is only partially exposed to the environment, as an extrusion between the tergites or sternites of living hosts. After fertilization, females are capable of producing many hundreds of thousands of active 1^st ^instar larvae who emerge from the cephalothorax.

Members of the strepsipteran lineage, Mengenillidae, are the sister-group to Stylopidia, a clade that includes all other extant Strepsiptera [[20], unpublished data]. This family represents a transitional phase in the evolutionary specialization of Strepsiptera, whereby both sexes leave the host before pupation, and females do not reproduce or release progeny in the unusual fashion outlined above. The level of simplification in free living mengenillid females is much less extreme than in Stylopidia (e.g. *X. vesparum*); legs, mouthparts and compound eyes are all still present, although strongly reduced. The invention of a completely endoparasitic female was probably one of the most important novelties leading to the radiation of this unique group [21]. Evolutionary studies attempting to unravel the developmental and ecological phenomena that make Strepsiptera so biologically interesting therefore require that species from before this important transition occupy a central role in research.

Insect mitochondrial genomes provide a useful medium to deepen and connect comprehension of microevolutionary forces of populations, like neutral drift and selective sweeps, to macroevolutionary events affecting species and/or deeper levels of divergence. The abundance of mitochondria in most metazoan cell and tissue types makes mtDNA an easily obtainable, universally plentiful marker, where a lack of introns or duplicate genes and non-coding variable spacer regions make amplification of mtDNA relatively uncomplicated [22] (although pseudogenes co-opted by the nucleus from the mitochondrial genome can create separate difficulties [23-26]). The near-complete sequence of the mt genome of *Xenos vesparum *is available, and as a member of the more specialized clade Stylopidia, it is important to place it into context. The mitochondrial genome of *Mengenilla australiensis *(Family Mengenillidae) was therefore chosen for sequencing. Genome arrangement, nucleotide content, codon usage and the secondary structure of ribosomal RNA genes are each comparatively assessed between two distant Strepsiptera, and more widely across Holometabola (a major division of insects defined by the process of complete metamorphosis, including the 4 very species-rich orders; Lepidoptera, Diptera, Hymenoptera, Coleoptera and 6 smaller orders).

## Results and discussion

### Genome composition

**Figure 1:**
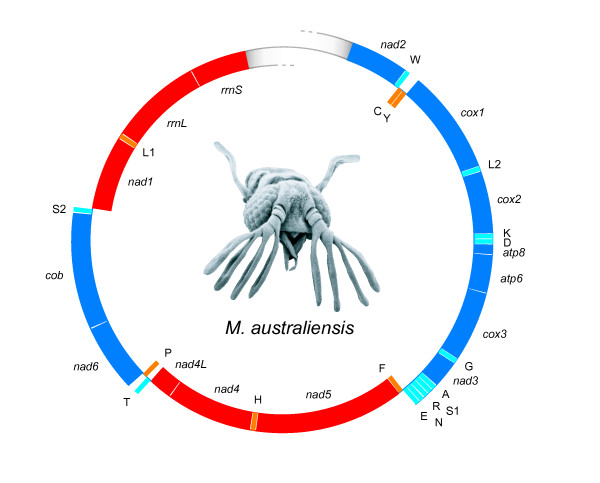
**Summary schematic of the mt genome of *M. australiensis***. Genes transcribed on the leading (α) strand are given in blue (PCGs) or light blue (tRNAs) and run clockwise. Genes of the opposite polarity are given in red (PCGs) or orange (tRNAs). Abbreviations are described in the text.

The near-entire mt genome fragment of *M. australiensis *is presented as a 13421 bp sequence, across which 13 protein-coding genes (PCGs), a large (16S) and a small (12S) subunit rRNA gene (*rrnL *and *rrnS *respectively) and 18 tRNA sequences can be identified (Figure 1). PCGs are in expected positions, but two tRNA translocations disrupt an otherwise ancestral genome arrangement. Serine_1 _(S1: AGN) moves to a position between Alanine (A) and Arginine (R), whereas Valine (V) is either lost, or transferred to a position in a region that spans the flanking region, potentially anywhere between tRNA-Ile and *nad2*. All start codons across the PCGs (*nad2 *is only partial) begin with the typical M- or I- residue [27], and end in TAA, TAG, TA, or T. The control region itself, including up to 4 flanking tRNA genes could not be amplified. A variety of approaches were explored, none of which were successful in spanning the presumed A+T region. Similar difficulties were encountered during the amplification of the mt genome of *X. vesparum *[28]. In our study, genome-specific primers designed to span the A+T region also performed well when reverse compliment versions were implemented. It is hypothesized that the region is unusually long and/or too problematic for even advanced *Taq *polymerases to amplify, as a result of extreme repetitiveness or secondary structural folding issues (or both).

**Figure 2:**
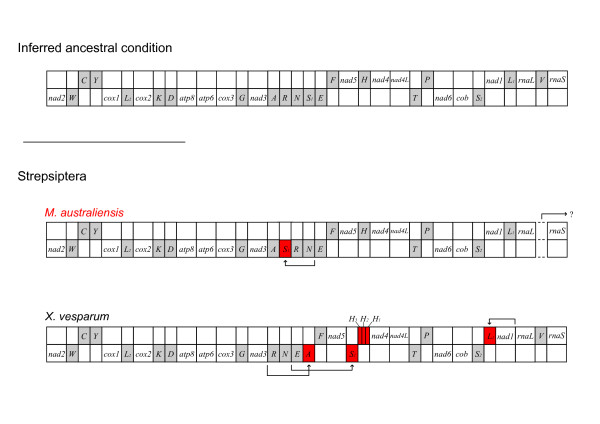
**Linear comparison of partial mt genome structure in *M. australiensis *and *X. vesparum***. Elements in red denote deviations from the ancestral holometabolan arrangement [29,30]. Readers are referred to the primary research for a complete catalogue of genome rearrangements in Holometabola: Hymenoptera [32]; Coleoptera [88-93]; Neuropterida [34,94,95], a shift from WCY to CWY is probably synapomorphic for Neuroptera but not for other neuropterid orders; Lepidoptera [33,75-81] are characterized by a synapomorphic tRNA rearrangement MIQ, from IQM, 5' of *nad2 *(not shown); Diptera [60-74].

Figure 2 compares the genome organization of *M. australiensis *and *X. vesparum *against the inferred ancestral arrangement [29,30]; the former having just two gene order rearrangements, compared to 4 in the latter. In the context of variation across a dataset containing 68 mitochondrial genomes taken from 6 major holometabolan groups compiled from Genbank (see methods), this would be consistent with a mostly ancestral arrangement being retained between the origins of the major holometabolan radiations, since every order contains representative ancestral (or near-ancestral as in *M. australiensis*) genome arrangements. Within certain orders, especially Hymenoptera (and certain hemimetabolous groups not discussed here [31]), lineages have undergone significant independent modifications (see [32] for a full review of hymenopteran mt genome evolution). Some synapomorphic alterations to this ancestral arrangement also exist in certain orders. These include a tRNA arrangement shift from IQM to MIQ across Lepidoptera [33] (outside of the fragment shown in Figure 2), and a likely shift from WCY to CWY in Neuroptera - a modification that is not shared by the other neuropterid orders [34]. But largely, the 68 genomes corroborate a hypothesis positing that the holometabolan orders emerged during a (relatively) brief period of ancient rapid radiation. Or in the context of phylogenetic tree shape, as an initial phase of short internal nodes, during which there would be insufficient time for changes in mitochondrial genome organization to become fixed between orders, followed by a longer period of external node expansion [35] in which enough time would pass for modifications to emerge independently along multiple branches within and between orders. With the inclusion of *M. australiensis*, whose genome does not share any of the alterations of *X. vesparum*, and is more representative of an ancestral insect arrangement, this also appears consistent for Strepsiptera: although greater sampling is required for a general picture of mt genome structural evolution to emerge.

**Figure 3:**
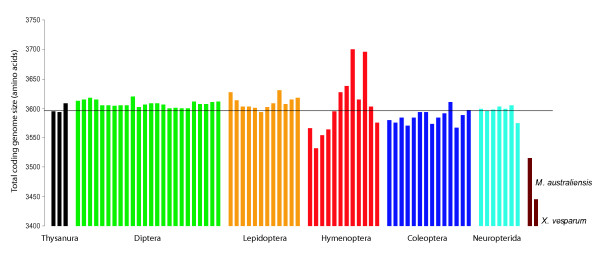
**Comparison of the total mt coding genome size (in amino acids) across Holometabola**. Black: Thysanura; Green: Diptera; Orange: Lepidoptera; Red: Hymenoptera; Blue: Coleoptera; Turquoise: Neuropterida; Burgundy: Strepsiptera. Each bar represents one species' genome. The superimposed line indicates the mean coding genome size across all 68 taxa.

The lengths of PCGs are truncated in both strepsipteran species, and especially so in *X. vesparum*. Across the 68 holometabolan dataset, *M. australiensis *has a reduction in mean content of 6.2 amino acids per gene. In *X. vesparum*, this reaches 11.8 amino acid deletions per gene. *X. vesparum *has the shortest *nad2*, *cox1*, *cox2*, *atp8*, *atp6*, *cox3*, *nad3 *and *nad4 *gene (Additional file 1), representing a 4.3% loss in amino acids from the average holometabolan complement. Its total coding genome is shorter by 154 amino acids, the next shortest is *M. australiensis *(81) followed by *Bombus ignitus *(67) and *M. bicolor *(45) (Figure 3). Given that substantial loss of gene content might be expected to severely compromise gene functionality and efficiency, it would be of considerable interest to investigate whether this kind of genomic streamlining is related in any way to the peculiar lifestyle of small endoparasitoid insects. In particular, does bottlenecking in strepsipteran populations lead to slightly deleterious mutations, like codon deletions, being fixed through random drift? Strepsipteran metapopulations could be experiencing the extreme extinction-recolonization population dynamics required for low effective population sizes to become influential, but there are no *a priori *reasons to suspect these should be so different from other hymenopteran host-parasitoid systems that depend on similar insect host groups (e.g. *Evania appendigaster*, *Venturia canescens*; both sampled in this study). Alternatively, bottlenecking could result from the population dynamics of mitochondria themselves, through very low numbers of these organelles being passed via the germ line of strepsipteran eggs, which are known to be extremely small [36].

Analyses to date do not reveal general patterns of mitochondrial genome evolution across holometabolan parasitic lineages [37,38], but these have concentrated largely on the comparative analysis of genome organization (gene order/orientation) itself. Within orders like Hymenoptera, although a correlation between the extent of mt genome modification and parasitism appears to be lacking [39,40] (with an additional implication that the position of mt genes might largely be neutral [32]), the truncation of PCGs as presented in this report, probably represents a separate issue. Determining the extent to which PCG truncation occurs within Strepsiptera must be a target for future investigations. Unfortunately, direct measures of insect mitochondrial effective population size have not yet been investigated.

**Figure 4:**
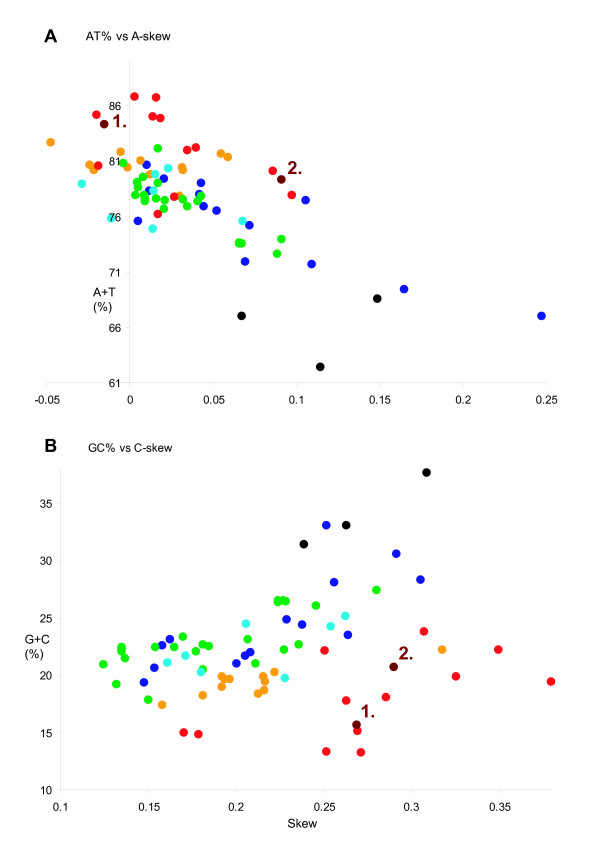
**Graphical summary of nucleotide content across 68 holometabolan mt genomes**. Measured in bp percentage (Y-axis) and level of nucleotide skew (X-axis) (described in methods, following [41]). Green: Diptera; Orange: Lepidoptera; Red: Hymenoptera; Blue: Coleoptera; Turquoise: Neuropterida; Burgundy: Strepsiptera. 1 and 2 refer to *M. australiensis *and *X. vesparum *respectively. A) A+T% vs A-skew B) G+C% vs C-skew. 3 genomes from Thysanura (in black), the host group for *M. australiensis *are also shown (see methods).

Overall nucleotide composition is typically A+T rich in *M. australiensis *(84.3%; 6^th ^highest amongst holometabolan insects), and like in *X. vesparum*, C-skew is high (+0.27). A scatter of the variation in skew and A+T% across the 68 holometabolan genomes (plus 3 thysanuran genomes) is shown in Figures 4A and 4B. Notably, in Figure 4B, of the most C-skewed and G+C% poor genomes (occupying the lower right quarter of this graph), 9 (of 12) data points are hymenopteran, 2 (of 2) are strepsipteran and 1 (of 12) is lepidopteran. A similar graph that including hemimetabolous insects [41] shows that only 1 other genome (*Schizaphis graminums*; Aphidoidea, 1 of 11 hemipteran genomes) is so C-skewed and A+T% rich. Conversely, the thysanuran genomes and a subset of Coleoptera are very A-skewed and distinctly more A+T% balanced (Figure 4A). This pattern is discussed in more detail in the following section.

### Codon Usage

**Figure 5:**
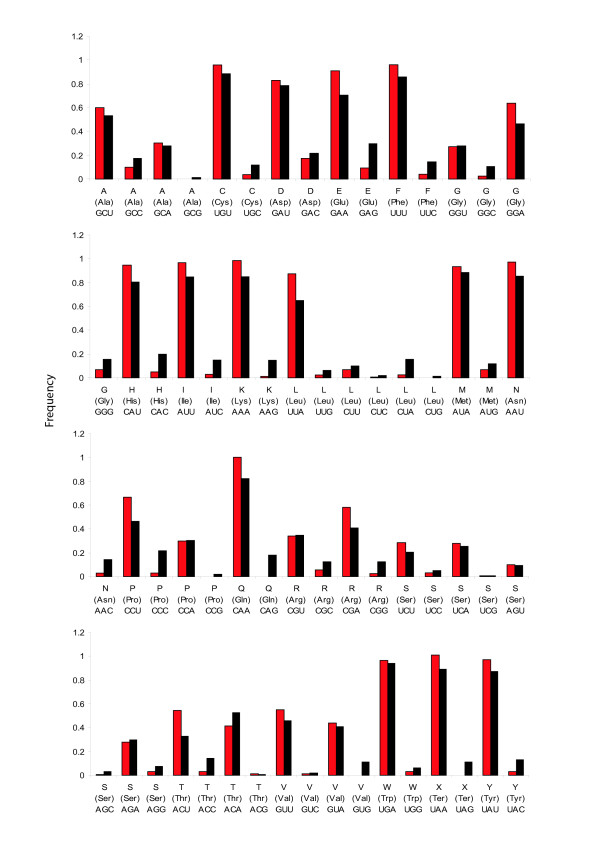
**Codon usage in *M. australiensis *(red) and *X. vesparum *(black)**. Codon frequencies are given (out of 1) for individual amino acids.

**Figure 6:**
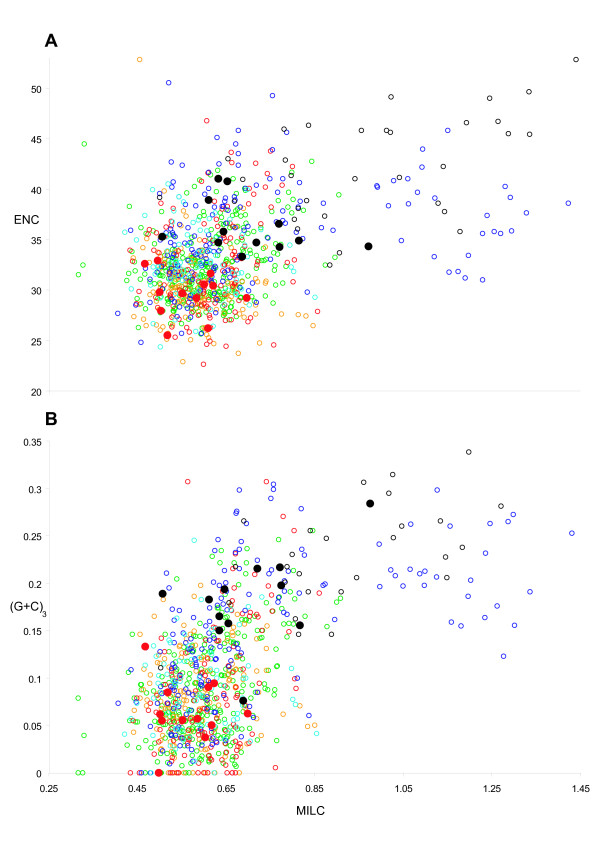
**Evaluation of codon bias across Holometabola**. A) Codon bias in *M. australiensis *(red filled circle) and *X. vesparum *(black filled circle) measured using ENC and MILC indices (described in methods), are compared against remaining Holometabola, whose data points are given as empty circles: the colour scheme follows taxonomic group as in Figure 3 and 4. Data points refer to individual PCGs. B) MILC values are compared against (G+C)_3_: which is G+C content at the 3^rd ^codon position of each PCG. This serves as an approximate measure of background nucleotide composition across individual PCGs.

PCGs from Strepsiptera and the holometabolan dataset were imported into INCA v2.1 [42] to analyze codon usage. *M. australiensis *and *X. vesparum *are compared in Figure 5: codon bias is significantly relaxed in *X. vesparum *(2-tailed paired T-Test of ENC/MILC vectors (see methods); P < 1 × 10^-5^), and an apparent switch in threonine (T) preference from ACU to ACA has occurred. In Figure 6A, these levels are assessed in context, alongside the holometabolan dataset using the ENC and MILC measures of codon bias for individual PCGs (see methods; [43]). In *X. vesparum*, all 12 PCGs occupy a much more relaxed, spread apart region of ENC/MILC space (the higher the value, the less codon bias), with average gene ENC and MILC values of 36.26 and 0.71 respectively. In *M. australiensis *these are 29.63 and 0.56, and in Holometabola, they average 32.77 (ENC) and 0.62 (MILC) over all genes. It follows that the values of codon bias among PCGs in *M. australiensis *should be found in the densest cluster of background holometabolan PCGs. The thysanuran genomes (empty black circles), alongside three coleopteran genomes (forming the isolated cluster of empty blue circles with MILC values > 1) have particularly balanced codon usage. Although beetles generally follow this rule; *Pyrophorus divergens, Tetraphalerus bruchi *and *Tribolium castaneum *in particular, have unusually high MILC values. Interestingly, unlike the thysanurans, these do not result in correspondingly elevated ENC values, possibly because of differences in the way MILC and ENC estimate bias [43].

These results are consistent with prevailing neutral mutational theories positing that genomic G+C content is the most significant factor in determining codon bias between organisms [44-47]. This may explain why *X. vesparum *has significantly relaxed ENC-MILC values, whose global G+C content is 5% higher than *M. australiensis*. For the thysanuran and beetle genomes, although G+C content in the 3^rd ^codon position of *individual *PCGs is not necessarily elevated (Figure 6B), it is global G+C% content that appears to matter: it has been documented that codon bias can be accurately predicted from intergenic regions [44], and it is unsurprising that these genomes should also have the highest genome wide G+C content across the 68 Holometabola dataset (Additional file 2). It is noticeable that the coleopteran *T. bruchi *has a very A-skewed genome (Figure 4A), which could also partly explain some of the discrepancies appearing between the different methods.

### Secondary structure of ribosomal and transfer RNAs

**Figure 7:**
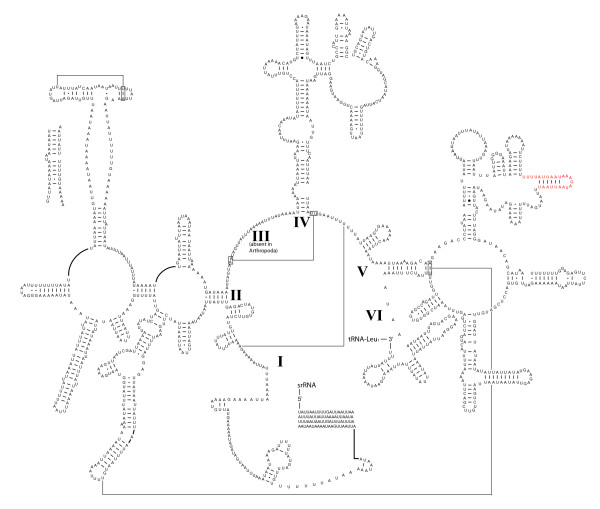
**Predicted secondary structure of the *rrnL *in *M. australiensis***. Regions in red receive little comparative support and remain ambiguous. Canonical A-U and G-C base pairings are given as '-'. G-U, G-A, and other non-canonical bp combinations are denoted as, '•', '○' and '●' respectively. Predicted tertiary interactions are represented by a line. Missing data are coded as 'N'.

**Figure 8:**
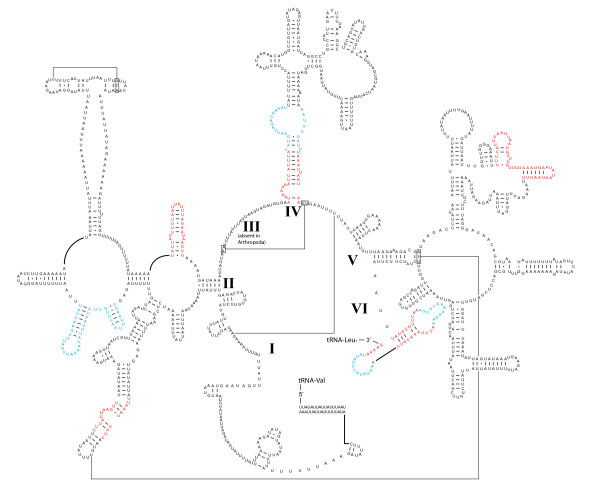
**Revised secondary structural prediction of the *rrnL *in *X. vesparum***. Regions in red receive little comparative support and remain ambiguous; regions in blue are structural predictions which have improved with the inclusion of comparative data from *M. australiensis*. Structural annotations follow figure 7.

With the addition of *M. australiensis*, a comparative revision of strepsipteran mitochondrial rRNA secondary structure was possible. Figures 7 and 8 are complete models for the *rrnL *in *M. australiensis *and *X. vesparum *respectively. The addition of new comparative evidence (from *M. australiensis *and *Apis mellifera *[48]) since Carapelli *et al*. (2006, [28]) enables some improvement over the structural model for *X. vesparum*. These modifications are highlighted in blue. Regions in which sequence variability is still too high for good structural covariation are highlighted in red. Overall, *M. australiensis *predictions are largely consistent with current insect consensus models but substantial portions of secondary structure remain problematic in *X. vesparum*. For example, the helix at the base of domain IV in *M. australiensis *is supported by comparative evidence from *Drosophila melanogaster *and *Apis mellifera*. In *X. vesparum*, most of this primary sequence in this helix is highly divergent and covarying base-pairing cannot be identified. Where this helix terminates in the single-strand bulge however, the base pairing AUG-UAU is supported by *M. australiensis *(AAG-UUU) which provides additional corroborative support for the bps in *D. melanogaster *and *A. mellifera*, which have, AAG-UUU and AGG-UCU respectively.

**Figure 9:**
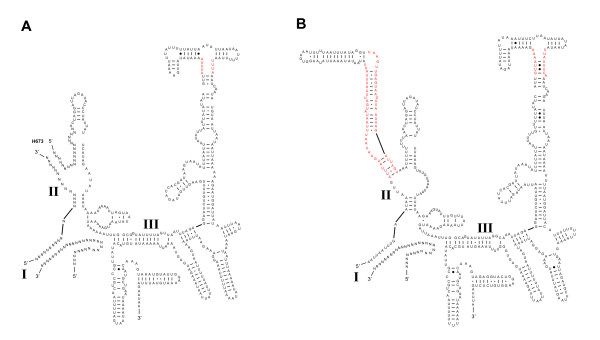
**Secondary structural predictions for the *rrnS *in Strepsiptera**. A) *M. australiensis*, B) *X. vesparum*. Regions in red receive little comparative support and remain ambiguous. Structural annotations follow figure 7.

**Figure 10:**
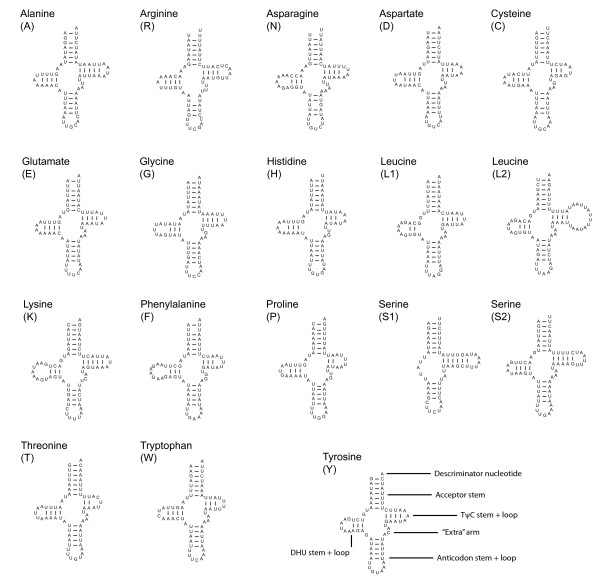
**Secondary structural predictions for *M. australiensis *tRNA genes**. Pertinent aspects relating to comparative structural differences between *X. vesparum *[28] and other insect lineages [39,41,58] are discussed in the main text.

*rrnS *secondary structural models for *M. australiensis *and *X. vesparum *are presented in Figure 9. Absence of sequence across domain I precludes a complete analysis, but partial models for domains II and III are generally conserved across Strepsiptera. Regions highlighted in red are more labile, and adequate consensus models for these structures are still lacking. The structural predictions for 18 tRNA genes are given in Figure 10. There is less scope for variation within extremely length-constrained tRNA genes. Despite this, the TψP stem is still too variable to be useful in a deep comparative framework: even between *M. australiensis *and *X. vesparum*, base-pairings demonstrate little covariation. The three remaining stem-loop structures are more conserved across Strepsiptera, and more useful in a comparative structural context. Within these regions, *X. vesparum *demonstrates more substantial modifications to typical insect tRNA structure than *M. australiensis *- consisting largely of single base or base-pair substitutions/compensations. For example, the proximal base-pairs of the acceptor stem of tRNA-Pro (P) in *X. vesparum *appears as UCAG-CUGA. In *M. australiensis*, Lepidoptera (*Ochrogaster lunifer *[41]) and Hymenoptera (*Vanhornia eucnemidarum *[39]) it is CAAA-UUUG. Similarly, the proximal DHU stem consists of a canonical A-U base-pairing in *X. vesparum*, where in *M. australiensis *and other insect groups it appears as a non-canonical G-U. Further, the base-pairing C-G, found across disparate insect lineages is substituted by A-U in the proximal stems of the tRNA-Lys (K) acceptor and the tRNA-Glu (E) DHU helices of *X. vesparum*.

## Conclusions

The mitochondrial genome of *X. vesparum *displays a number of characteristics that are not shared by its distant relative *M. australiensis*. In the latter, only 2 tRNA translocations disrupt an otherwise ancestral insect genome organization, whereas in *X. vesparum*, 3 tRNA translocations and at least 2 duplications have occurred (Figure 2). Codon bias in *X. vesparum *is also significantly relaxed: *M. australiensis *occupies a much more typical region of ENC/MILC space (Figures 5 and 6). Further, rRNA secondary structural model predictions in *M. australiensis *are much more consistent with current insect consensus structures (Figures 7, 8, 9 and 10). In *X. vesparum*, increased variability at the primary sequence level precludes the assembly of well corroborated models in several parts of the rRNA genes. Both strepsipteran genomes are unusually C-skewed and A+T% rich. Interestingly, PCGs are noticeably truncated in both strepsipteran taxa, but especially so in *X. vesparum *which has lost nearly twice as many codons (154) as *M. australiensis *(81), equating to 4.3% and 2.2% fewer codons than the average holometabolan genome respectively.

These results provide useful insight into the evolution of strepsipteran mitochondria spanning the shift from a largely free-living insect into an extremely unique and simplified endoparasitoid, since the emergence of Strepsiptera over 100 million years ago [49]. The modifications to the mitochondrial genomes of *X. vesparum *and *M. australiensis *appear consistent with organisms that have evolved extremely derived lifestyles. *M. australiensis *represents a transitional phase in the evolutionarily specialization of Strepsiptera, and the composition, architecture and structure of its genome reflects this. These observations raise important questions about the changing selective environment in (mitochondrial) genomes belonging to small, and almost entirely host-dependent endoparasitoids. Future investigations into the evolutionary and developmental origins of this unique biological system must ensure that strepsipteran species from Mengenillidae are adequately sampled: wide sampling across this group is crucial if the underlying ancestral signal is to be maximized.

## Methods

### Specimen description

DNA was used from a small set of free-flying males collected in light traps, leaving little possibility for contamination through thysanuran host DNA. Nuclear and mitochondrial gene alignments from a widely sampled Strepsiptera taxon set (unpublished) confirm this. We also include 3 thysanuran genomes in Figure 8 to emphasize the genome composition differences between *M. australiensis *and its target host group. Specimens were collected on the 16^th ^March 2006 in Australia, Queensland, Blackdown Tablelands NP, South Mimosa Creek, 50 m dstr road, 794 mao. Coordinates as follows: S23 47.687'E 149 04. 195'. Light trap loc 34 (Collectors were N. Jönsson, T. Malm & D. Williams). Complete species name: *Mengenilla australiensis *Kifune & Hirashima 1983.

### Genome isolation and sequencing

*M. australiensis *specimens were extracted for PCR either by macerating tissue and digesting overnight at 50°C with Chelex 100 grade resin (Bio-Rad Cat. no 142-1253, Hemel Hampstead, UK) and enzyme Proteinase K (BIOLINE, Cat. no BIO-37037, London, UK) or by employing a QIAGEN blood and tissue column extraction protocol. Amplification of the *M. australiensis *mitochondrion was carried out by amplifying three ~3 kb fragments, with intervening sections that did not overlap being amplified by standard PCR. Long PCR mixes containing 1 μl BIO-X-ACT long DNA polymerase (BIOLINE, Cat. no. BIO-21049), 1 μl dNTPs, 25 μl 2× Polymate additive (BIOLINE, Cat. no. BIO-37041) for A+T rich sequences, 5 μl 10× opti-buffer, 4 μl MgCl_2 _solution, 3 μl forward/reverse primers and 5-10 μl of genomic DNA were made up to 50 μl reactions with ultrapure (NANOpure Diamond™) water. Long PCR cycling parameters were as follows: 92°C for 2 minutes; 10 cycles of: 92°C for 10 seconds, 53°C for 30 seconds and 68°C for 13 minute; 28 cycles of: 92°C for 15 seconds, 53°C for 30 seconds and 68°C for 14 minute; with an extension step of 7 minutes at 68°C. Long PCR fragments were gel extracted (QIAGEN Cat no. 28704, Hemel Hampstead, UK) and used as templates in subsequent sequencing reactions. Standard PCR mixes contained 0.5 μl *Taq *polymerase, 1 μl dNTPs, 5 μl MgCl_2 _free buffer, 4 μl MgCl_2_solution, 3 μl forward/reverse primers and 2 μl of genomic DNA, made up to 50 μl reactions with ultrapure (NANOpure Diamond™) water. Standard PCR cycling parameters were as follows: 94°C for 2 minutes, then 35 cycles of 94°C for 1 minute, *T*°C for 45 seconds (*T *varied with primer pair) and 72°C for 1 minute, with an extension step of 5 minutes at 72°C. For difficult regions, Phusion high fidelity *Taq *was utilized, following the manufacturer's instructions. Standard PCR products were cleaned using 2 μl of Shrimp Alkaline Phosphatase (SAP) and 3 μl of Exonuclease I (in a 1:10 SAP buffer dilution) per 50 μl PCR reaction, or fragments were extracted from agarose gels and sequenced directly. Problematic fragments that did not sequence directly were inserted into pGEM-T Easy plasmid vectors (Promega, Southampton, UK), and multiple clones were sequenced for each fragment. Sequencing reactions were performed using the BigDye^® ^Terminator v3.1 cycle sequencing kit with the following modifications: 1 μl of BigDye^®^, 1.5 μl 5× Buffer, 1 μl primer (3.2 pmol), 1-3 μl genomic DNA made up to 10 μl reactions with ultrapure (NANOpure Diamond™) water. Sequence reads were generated using an Applied Biosystems 3730xl DNA Analyzer. Sequences were imported into GridinSoft Notepad (Lite Edition; http://notepad.gridinsoft.com) and BioEdit (version 7.0.5.3; [50]) for analysis. Primer pairs employed are given in Additional file 3. The annotated genome fragment can be found in Genbank under the accession number GU188852. Voucher specimens and extractions are deposited in the Hope Entomological Collections, Oxford University Museum of Natural History, Oxford, UK, and the Swedish Museum of Natural History, Stockholm, Sweden.

### Genome charactarization

PCGs and their boundaries were diagnosed via sequence comparison across alignments derived from the 68 holometabolan genome dataset. Start codons found to be in-frame and not overlapping with upstream genes on the same strand were usually identified as 5' gene boundaries. Stop codons were typically identified as TAA, TAG, TA, or T. A complete annotation of the *M. australiensis *genome is given in Additional file 4. One non-coding fragment, 24 bp in length, was identified between the stop codon of *nad1 *and the beginning of tRNA-Ser_2_. Secondary structural predictions for ribosomal and transfer RNAs were ascertained by implementing a comparative structural framework [51-55] (for an online tutorial see the jRNA web site: http://hymenoptera.tamu.edu/rna/), in conjunction with a thermodynamic-based RNA folding algorithm (*mfold*; [56]). *mfold *gave initial raw estimates of structure: a tool that is especially useful for variable domains with highly divergent primary sequence. In this approach, structures with the lowest thermodynamic stability values were most highly considered (although structures with only marginal thermodynamic differences were also evaluated) and robust comparative evidence where available, took priority. The following ribosomal RNA structural templates from the Comparative RNA Web (CRW) site database [57] were used: *Escherichia coli*, *Drosophila melanogaster*, *Drosophila virilis*, *Chorthippus parallelus *and *Apis mellifera *[48]. For tRNA genes, the following models were used as comparative data points: *X. vesparum *(Strepsiptera: [28]), *O. lunifer *(Lepidoptera: [41]), *V. eucnemidarum *(Hymenoptera: [39]) and *Cryptopygus antarcticus *(Collembola: [58]), structures were verified for accuracy in tRNA-Scan [59]. Gene order was assessed across Holometabola using the inferred ancestral insect arrangement as a comparative standard [29,30]. As a measure of gene length fluctuation, total and individual gene length was counted across strepsipteran PCGs, and compared across the dataset of 68 holometabolan genomes. To include *nad2 *gene, the 5' portion (approx. 100 amino acids) was excluded from analysis so that *M. australiensis *could be assessed alongside the entire dataset.

The 68 holometabola mitochondrial genome dataset was compiled from previously published data across the 6 major holometabolan divisions: Diptera [60-74], Lepidoptera [33,75-81], Hymenoptera [39,82-87], Coleoptera [88-93], Neuropterida [34,94,95] and Strepsiptera [28].

### Nucleotide content and Codon usage

A+T% and G+C% values were calculated for the α strand of the *M. australiensis *genome, which runs clockwise in the direction of transcription from *nad2 *(Figure 1). Genomic A- and G-skew measures were calculated using total nucleotide % values in the following manner: [A-T]/[A+T] and [G-C]/[G+C] following Perna & Kocher [96]. A complete list of nucleotide content values for the 68 holometabolan dataset is given in Additional file 3. Codon usage was investigated by importing the complete cDNA datasets of 68 available holometabolan mitochondrial genomes, including *X. vesparum *and the sequenced genome of *M. australiensis *into INCA v2.1 [42]. Two independent measures of codon bias: ENC (Effective Number of Codons used) and MILC (Measure Independent of Length and Composition) are implemented, whose statistical justification and behaviour under simulation differ markedly [43]. These two independent measures were combined as a singe vector statistic as follows: √(ENC^2 ^+ MILC^2^). The shortest gene, *atp8 *was also excluded from analyses due to artificially increased ENC/MILC bias in genes shorter than around 100 amino acids. *nad4L *was of borderline length (approx. 90-105 amino acids) and was retained, although *nad4L *from *Drosophila sechellia *and *Melipona bicolor *produced arbitrary ENC 'cut-off' values of 61, because ENC behaves less stably at borderline gene lengths; these data points were removed. Readers are referred to [43] for a detailed explanation.

## Abbreviations

mt: mitochondrial; PCG: protein-coding gene; A+T region: the putative control region; α strand: leading strand of transcription; ENC: Effective Number of Codons used; MILC: Measure Independent of Length and Composition; *rrnL *and *rrnS*: large (16S) and small (12S) subunit ribosomal RNA (rRNA) gene; tRNA genes are denoted as single letter amino acid IUPAC-IUB abbreviations; *atp6 *and *atp8*: ATP synthase subunits 6 and 8; *cob*: apocytochrome b; *cox1*-*3*: cytochrome c oxidase subunits 1-3; *nad1*-*6 *and *nad4L*: NADH dehydrogenase subunits 1-6 and 4L; MRCA: Most Recent Common Ancestor.

## Competing interests

The authors declare that they have no competing interests.

## Authors' contributions

DPM and AH devised and completed laboratory work. DPM conducted analyses and early manuscript draughts. All authors contributed to the final version of the manuscript.

## Supplementary Material

Additional file 1 **Coding gene lengths**. Spreadsheet containing coding gene lengths (in amino acids) across the Holometabola dataset. Click here for file

Additional file 2 **Nucleotide content**. spreadsheet of genomic nucleotide content, A-skew and C-skew across the Holometabola dataset. Click here for file

Additional file 3 **Primers**. List of primer pairs used in the study. Click here for file

Additional file 4 **Gene annotation**. Detailed summary of genome fragment architecture. Click here for file
